# Molecular detection of Rickettsia and Borreliosis pathogens in the areas of Boumerdes and Tizi Ouzou (Algeria)

**DOI:** 10.1186/1742-4690-9-S1-P41

**Published:** 2012-05-25

**Authors:** Afif T Chaouche, I Bitam, K Amara, I Yahiaoui

**Affiliations:** 1 Microbiologie at Université, Tlemcen, Algeria

## Aim

Mediterranean spotted fever, and the Lyme disease are emerging infectious diseases with significant impact on public health, a study of their detection methods and transmission modes is very useful. As vector diseases, this study consist of finding the agents in question on ticks (vectors of these diseases) in the area of Boumerdes and Tizi-ouzou, coastal areas in central Algeria, and checking the transmission of these germs from generation of ticks to another.

## Materials and methods

The investigation launched from December 2009 to June 2010, has raised ticks on dogs and cows in these areas, and after the identification of genus and species of the 182 collected ticks, 56 of them were analyzed by PCR followed by electrophoresis. The detection of Rickettsia was also done by the Gimenez stain from a drop of hemolymph of a tick collected on a slide. A breeding of ticks has been launched to track their life cycle and eventual transmission of Rickettsia by ovarian trans-staidly.

## Results

11 of 56 ticks were found carrying Rickesttsia conorii conorii, (the bacteria responsible for Mediterranean spotted fever), and no ticks carrying Borrelia Burgdorferi (bacteria that causes Lyme disease) was found. After analyzing the ticks of the first and second generation, the presence of these bacteria has been found.

## Conclusion

The results of this investigation allow confirm the presence of Rickettsial diseases in the regions of Boumerdes and Tizi Ouzou, but also the transmission from one generation of germs on ticks, the risk of transmission to humans is certain in case of tick bite, from which the necessity of the vector control. 1. Sahibi H. & Rhalem A. (2007) : Tiques et maladies transmises par les tiques chez les bovins au Maroc, MADER/DERD N°151 2. Doudier B., Pages F., Parola P., Socolovschi C., Tiques et maladies transmises à l’homme en Afrique, Médecine Tropicale, Med Trop 2008, 68, 119-133 3. Bitam I., (2008) : Rickettsioses associées aux arthropodes, la revue medicopharmaceutique N°48-3éme trimester, p38-39.

